# Associations of ficolins and mannose-binding lectin with acute myeloid leukaemia in adults

**DOI:** 10.1038/s41598-020-67516-2

**Published:** 2020-06-29

**Authors:** Anna Sokołowska, Anna S. Świerzko, Gabriela Gajek, Aleksandra Gołos, Mateusz Michalski, Mateusz Nowicki, Agnieszka Szala-Poździej, Anna Wolska-Washer, Olga Brzezińska, Agnieszka Wierzbowska, Krzysztof Jamroziak, Marek L. Kowalski, Steffen Thiel, Misao Matsushita, Jens C. Jensenius, Maciej Cedzyński

**Affiliations:** 1 Laboratory of Immunobiology of Infections, Institute of Medical Biology, Polish Academy of Sciences, Lodowa 106, 93-232 Lodz, Poland; 2 Department of Hematology, Institute of Hematology and Transfusion Medicine, I. Gandhi 14, 02-776 Warsaw, Poland; 3 Department of Hematology, Copernicus Memorial Hospital in Łódź Comprehensive Cancer Center and Traumatology, Pabianicka 62, 93-513 Lodz, Poland; 4 Department of Hematology, Medical University of Łódź, Ciołkowskiego 2, 93-510 Lodz, Poland; 5 Department of Immunology and Allergy, Medical University of Łódź, Pomorska 251, 92-213 Lodz, Poland; 6 Department of Rheumatology, Medical University of Łódź, Pieniny 30, 92-003 Lodz, Poland; 7 Department of Biomedicine, Aarhus University, Høegh-Guldbergs Gade 10, 8000 Aarhus C, Denmark; 8 Department of Applied Biochemistry, Tokai University, 4-1-1 Kitakaname, Hiratsuka, Kanagawa 259-1292 Japan

**Keywords:** Immunology, Biomarkers, Risk factors

## Abstract

We investigated clinical associations of ficolins and mannose-binding lectin (MBL) in 157 patients suffering from acute myeloid leukaemia (AML). Concentrations of ficolin-1, ficolin-2, ficolin-3 and MBL (before chemotherapy) in serum were determined as were selected polymorphisms of the corresponding genes (*FCN1, FCN2, FCN3* and *MBL2*). The control group (C) consisted of 267 healthy unrelated individuals. Median level of ficolin-1 in patients was lower (*p* < 0.000001) while median levels of ficolin-2, ficolin-3 and MBL were higher (*p* < 0.000001, *p* < 0.000001 and *p* = 0.0016, respectively) compared with controls. These findings were generally associated with AML itself, however the highest MBL levels predicted higher risk of severe hospital infections (accompanied with bacteremia and/or fungaemia) (*p* = 0.012) while the lowest ficolin-1 concentrations tended to be associated with prolonged (> 7 days) fever (*p* = 0.026). Genotyping indicated an association of *G/G* homozygosity (corresponding to *FCN1* gene − 542 *G* > *A* polymorphism) with malignancy [*p* = 0.004, OR = 2.95, 95% CI (1.41–6.16)]. Based on ROC analysis, ficolin-1, -2 and -3 may be considered candidate supplementary biomarkers of AML. Their high potential to differentiate between patients from non-malignant controls but also from persons suffering from other haematological cancers (multiple myeloma and lymphoma) was demonstrated.

## Introduction

Acute myeloid leukaemia (AML) is the most common leukaemia affecting adults (approximately 80%; > 1% of total cancers). It is an aggressive malignancy, characterized by clonal proliferation and accumulation of morphologically and functionally immature blast cells, originating from progenitor haematopoietic cells after neoplastic transformation. It is associated with impairment of haematopoiesis resulting in anaemia, high susceptibility to infections and haemorrhage. AML more often affects males. It may develop at any age but morbidity is known to increase in older people^1–5^. The remission-inducing chemotherapy leads to bone marrow aplasia, causing anaemia, neutropenia and thrombocytopenia. During the period of aplasia (2–3 weeks), patients have to be carefully monitored and receive supportive medication, including blood products and antibiotics. The severe neutropenia contributes to the enhanced susceptibility to infections and high mortality at this stage^6–9^. Twenty-eight % of AML patients survive for 5 years (www.seer.cancer.gov).

Collectins and ficolins are collagen-related, multimeric lectins acting as pattern-recognising molecules, involved in anti-microbial and anti-cancer immunity. All human ficolins (ficolin-1, -2, -3) and some collectins (including mannose-binding lectin, MBL) form complexes with mannose-binding lectin-associated serine proteases (MASP) which enables them to activate the complement system via the lectin pathway^10,11^. They are also able to opsonize pathogens and abnormal host cells. Therefore, they may prevent certain infections or development of some malignancies via direct lysis of cells expressing pathogen/danger-associated molecular patterns or by enhancement of phagocytosis^12–15^. On the other hand, complement-activating lectins may be involved in excessive or chronic inflammation which may be harmful to the host, leading in some cases to organ failure or carcinogenesis, respectively^16–18^.

The aims of this study were to investigate possible associations of ficolins and/or MBL levels in sera taken before starting chemotherapy with acute myeloid leukaemia itself and with susceptibility to hospital infections after chemotherapy. We also aimed to investigate the possible associations with selected polymorphisms of the genes encoding for these proteins. Concentrations of ficolin-1, ficolin-2, ficolin-3 and MBL in sera (before chemotherapy) were determined as were selected single nucleotide polymorphisms (SNP) of the corresponding genes (*FCN1, FCN2, FCN3* and *MBL2,* respectively). Totally, 15 SNP, affecting expression of genes, concentration and/or activity of their products were chosen. The variant (*A*) alleles corresponding to − 542 *G* > *A* (rs10120023) and − 144 *C* > *A* (rs10117466) *FCN1* gene polymorphisms are associated with higher expression of specific mRNA in monocytes and granulocytes as well as higher ficolin-1 serum level^19,20^. In contrast, presence of *A*, *C*, *G* variants, related to + 6,658 *G* > *A* (Ala218Thr, rs148649884), + 7,895 *T* > *C* (Ser268Pro, rs150625869 and + 7,959 *A* > *G* (Asn289Ser, rs138055828) SNP, respectively, results in lower ficolin-1 concentration. Furthermore, exchange of amino acid residues at positions 218 and 289 affects recognition of ligands while *C/C* homozygosity at position + 7,895 was hypothesized to result in total ficolin-1 deficiency^19^.

In the case of *FCN2* gene, we selected two pairs of polymorphisms in strong linkage disequilibrium. The first, − 64 *A* > *C* (rs78654553) and + 6,424 *G* > *T* (Ala258Ser, rs7851696) is associated with relatively low ficolin-2 serum levels in carriers of minor alleles. The second one, − 4 *A* > *G* (rs17514136) and + 6,359 *C* > *T* (Thr236Met, rs17549193), has the opposite effect^21–23^. The variant alleles at positions + 6,359 and + 6,424 were moreover demonstrated to influence ligand binding capacity of the protein^21^.

A frameshift mutation of the *FCN3* gene (+ 1637*delC,* rs28357092) leads to the rare total ficolin-3 deficiency in variant homozygotes and low levels of this protein in sera of heterozygotes^24^. Single nucleotide polymorphisms of the *MBL2* gene promoter region: − 550 *G* > *C* (rs11003125, usually called *H/L*) and − 221 *C* > *G* (rs7096206, *Y/X*) influence MBL serum concentration. Coding region SNP: + 223 *C* > *T* (Arg52Cys, rs5030737), + 230 *G* > *A* (Gly54Asp, rs1800450) and + 239 *G* > *A* (Gly57Glu, rs1800451), known as *A* > *D, A* > *B* and *A* > *C* (their variant alleles are commonly designated *O*) affect both MBL level and activity. The presence of *O* alleles is associated with diminished opsonic properties and complement activation, due to impaired oligomerization of the molecule and ability to form complexes with MASP. The increased sensitivity to endogenous metalloproteases contributes in turn to lower MBL concentration. As strong linkage disequilibria exist between the afore-mentioned SNP [and another one, not studied here: + 4 *C* > *T* (rs7095891, *P/Q*, in *MBL2* gene 5′-untranslated region)], seven haplotypes only are considered relatively common: *HYPA, LYPA, LYQA, LXPA, HYPD, LYPB, LYQC* (reviewed in^10^).

Although the uncommon missense variants of *FCN1* (rs148649884, rs150625869, rs138055828) as well as the frameshift mutation of *FCN3* (rs28357092) are relatively rare^19,24^, they markedly influence concentration and/or function of their corresponding proteins. Therefore we supposed that those SNP may modulate the susceptibility both to AML itself and to related hospital infections, and their effects would be strong enough to be detected.

## Results

### *FCN1* gene polymorphisms and serum concentrations of ficolin-1

All patients and controls were *T/T* and *A/A* homozygotes for + 7,895 *T* > *C* (rs150625869) and + 7,959 *A* > *G* (rs138055828) *FCN1* gene polymorphisms, respectively. The *G/G* genotype (− 542 *G* > *A* SNP, rs10120023) was more common among patients compared with controls (C group) [*p* = 0.018, OR = 1.6, 95% CI (1.1–2.45)] (Table 1). That reflected mainly relative high number of *G/G* homozygous patients who had no infective complications during 4-week hospital stay. After multiple logistic regression analysis and correction for multiple comparisons, the difference between patients and controls remained significant [*p* = 0.004, OR = 2.95, 95% CI (1.41–6.16), corrected for sex and age, dominant model] (Table 1). It should be stressed that in contrast to controls, distribution of alleles among patients did not comply with Hardy–Weinberg equilibrium: *G/G* homozygosity was more common than predicted (*p* = 0.015). No significant differences between AML and C groups were found for − 144 *C* > *A* (rs10117466) or + 6,658 *G* > *A* (rs148649884) polymorphisms. However, *A/A* homozygosity for the first mentioned was more common among patients who developed bacteremia/fungaemia (AML-A) than among those with no such complications (AML-D) [*p* = 0.01, OR = 6.89, 95% CI (1.4–33.98)]. After reconstruction of haplotypes with the EM maximum-likelihood method (Supplementary Table 1), we found the *GCG* haplotype (corresponding to − 542 *G* > *A*, − 144 *C* > *A* and + 6,658 *G* > *A* SNP, respectively) to be the most common in all groups. Its estimated frequency was however significantly lower among patients who experienced infections with bacteremia/fungaemia, compared not only with healthy controls but also with patients who had no hospital infections (*p* < 0.01; Supplementary Table 1).

**Table 1:** Frequency of genotypes associated with selected *FCN1* single nucleotide gene polymorphisms.

Polymorphism	Genotype	Group					
		C	AML	AML-A	AML-B	AML-C	AML-D
-542 *G* > *A*	*G/G*	95 (37)	77 (49)^1^	19 (46.3)	26 (44.1)	5 (83.3)	27 (52.9)^2^
(rs10120023)	*G/A*	125 (48.6)	54 (34.4)	13 (31.7)	24 (40.7)	1 (16.7)	16 (31.4)
	*A/A*	37 (14.4)	26 (16.6)	9 (22)	9 (15.3)	0	8 (15.7)
-144 *C* > *A*	*C/C*	98 (38.1)	64 (48.8)	10 (24.4)	26 (44.1)	1 (16.7)	27 (52.9)
(rs10117466)	*C/A*	123 (47.9)	72 (45.9)	22 (53.7)	25 (42.4)	3 (50)	22 (43.1)
	*A/A*	36 (14)	21 (13.4)	9 (22)^3^	8 (13.6)	2 (33.3)	2 (3.9)
+ 6,658 *G* > *A*	*G/G*	256 (99.6)	156 (99.4)	41 (100)	58 (98.3)	6 (100)	51 (100)
(rs148649884)	*G/A*	1 (0.4)	1 (0.6)	0	1 (1.7)	0	0
	*A/A*	0	0	0	0	0	0

Percentages are shown in parentheses. Allele distributions were in accordance with Hardy–Weinberg equlibrium, with an exception for − 542 *G* > *A* polymorphism in AML group where G/G homozygosity was more common than predicted.

C: controls; AML-A: patients who experienced infections with proven bacteremia and/or fungaemia; AML-B: patients who experienced infections with no bacteremia; AML-C: patients who experienced febrile neutropenia; AML-D: patients who experienced none of afore-mentioned complications within 4 weeks of hospital stay.

^1^*p* = 0.018, OR = 1.6, 95% CI (1.1–2.45) (vs. C) [multiple logistic regression, dominant model: *p* = 0.004, OR = 2.95, 95% CI (1.41–6.16)].

^2^*p* = 0.041, OR = 1.9, 95% CI (1.05–3.51) (vs. C).

^3^*p* = 0.01, OR = 6.9, 95% CI (1.4–33.98) (vs. AML-D).

In contrast, *ACG* and *GAG* haplotypes were commoner in the AML than in the C group. The last mentioned was particularly frequent within the AML-A group (significant difference in comparison with C as well as AML-B groups). Furthermore, the frequency of both *ACG* and *GAG* variants was higher in AML-D in comparison with the control group while an inverse relationship (also in the case of combined AML group) was found for the *AAG* variant (Supplementary Table 1).

**Figure 1:**
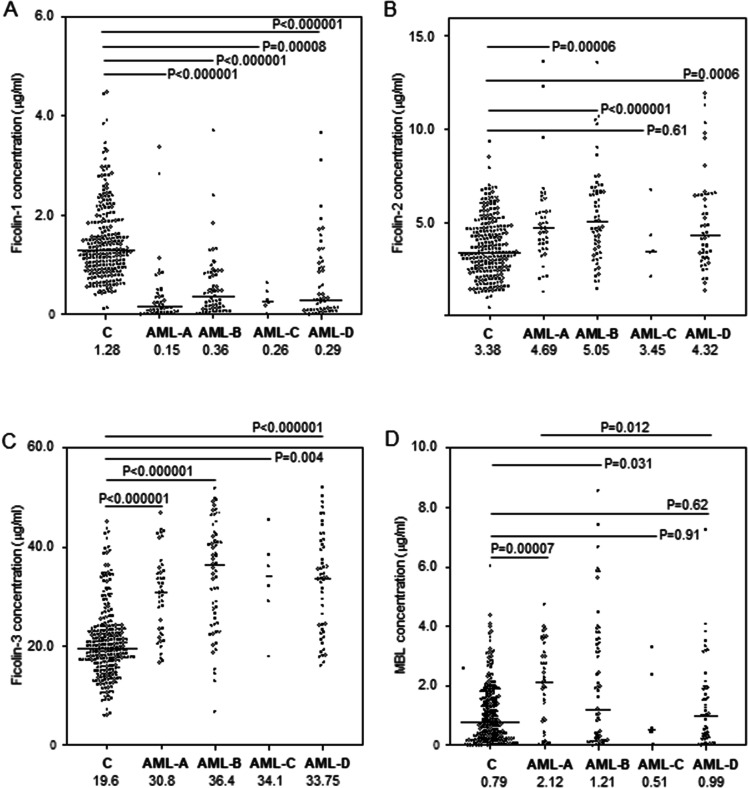
Serum concentrations of ficolin-1 (**A**), ficolin-2 (**B**), ficolin-3 (**C**) and mannose-binding lectin (**D**) in patients (before chemotherapy) and controls. Bars present median values (given below group descriptions). C: controls; AML-A: patients who experienced infections with proven bacteremia and/or fungaemia; AML-B: patients who experienced infections with no bacteremia; AML-C: patients who experienced febrile neutropenia; AML-D: patients who experienced none of afore-mentioned complications within 4 weeks of hospital stay. The medians of protein concentrations were compared using the Mann–Whitney *U*-test.

Median serum ficolin-1 concentration in AML patients before starting chemotherapy was almost fivefold lower than in healthy controls (260 ng/ml vs. 1,277 ng/ml; *p* < 0.000001, Mann–Whitney *U*-test), irrespective of complications recorded during hospital stay or *FCN1* genotype (Fig. 1A, 2A, B). The lowest median before starting treatment was noted in patients who suffered from hospital infections accompanied by bacteremia/fungaemia after chemotherapy (151 ng/ml) (Fig. 1A). Furthermore, lower ficolin-1 before chemotherapy seemed to predict prolonged fever: the median for patients who experienced fever for > 7 days was as low as 119 ng/ml while for the other patients, it equalled 284 ng/ml (*p* = 0.026). However, that result was not statistically significant after Bonferroni correction. Furthermore, there was no association with mortality.

**Figure 2:**
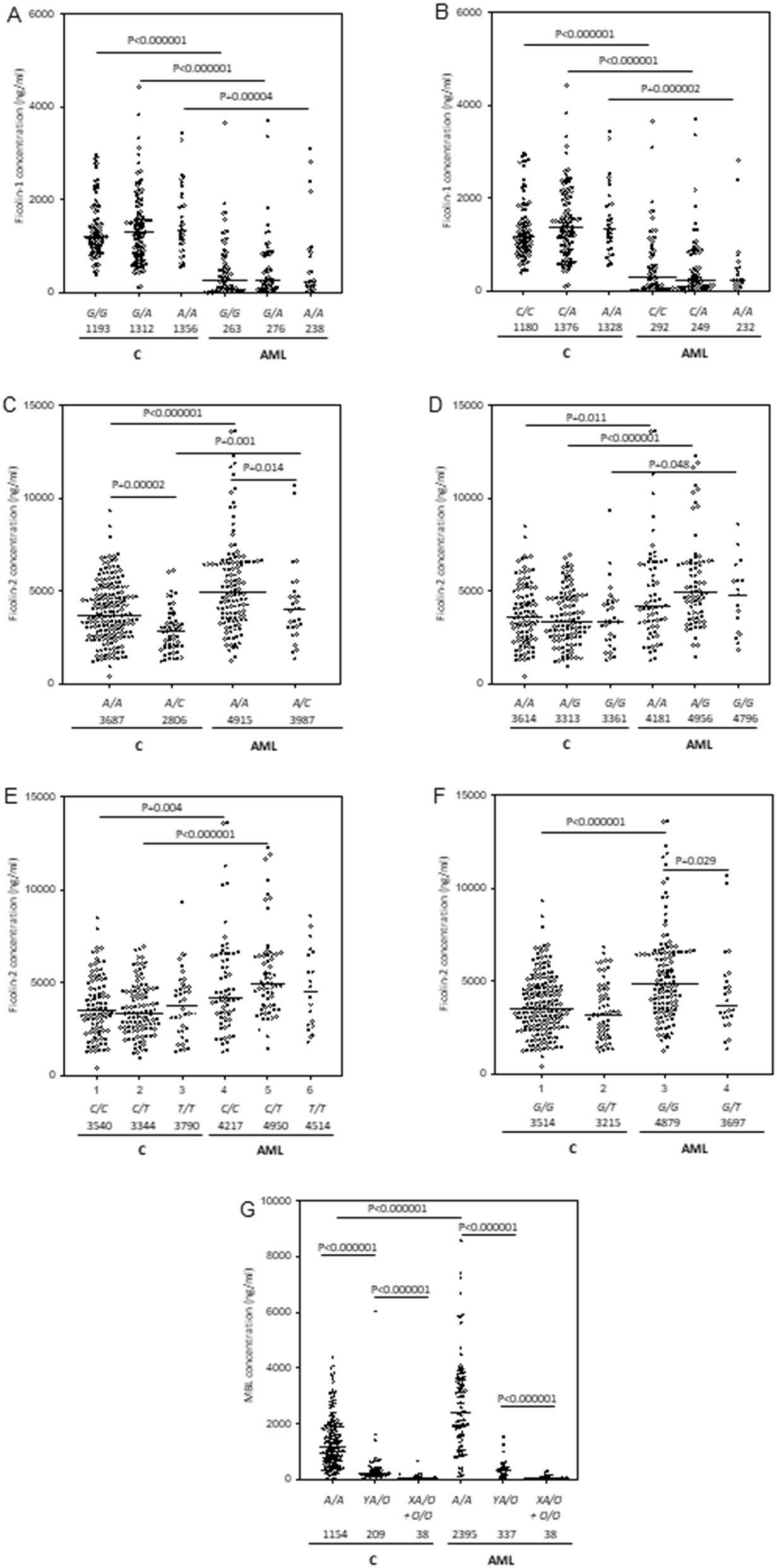
Serum concentrations of ficolin-1, ficolin-2 and mannose-binding lectin in patients (AML) and controls (C), depending on polymorphisms investigated. Bars represent median values (given below genotype descriptions). The medians were compared using the Mann–Whitney *U*-test (p given when significant differences were observed). (**A**) ficolin-1 levels and − 542 *G* > *A* polymorphism; (**B**) ficolin-1 levels and − 144 *C* > *A* polymorphism; (**C**) ficolin-2 levels and − 64 *A* > *C* polymorphism; (**D**) ficolin-2 levels and − 4 *A* > *G* polymorphism; (**E**) ficolin-2 levels and + 6,359 *C* > *T* polymorphism; (**F**) ficolin-2 levels + 6,424 *G* > *T* polymorphism. (**G**) MBL levels and common promoter (*Y/X*) and exon 1 (*A/O*) polymorphisms.

Low ficolin-1 levels (< 620 ng/ml) were therefore very common among patients with AML (71.8%) in comparison with healthy controls (10.2%) [*p* < 0.00001, OR = 23.4, 95% CI (13.64–40.2)]. The difference remained highly significant after multiple logistic regression analysis and Bonferroni correction [*p* < 0.001 OR = 38.2 95% CI (17.57–83.04)]. Consequently, the highest frequency (82.9%) of low ficolin-1 was associated with the most severe infections [*p* < 0.000001, OR = 44.7, 95% CI (17.95–111.3) vs. C group]. However, concentrations < 620 ng/ml were often noted in other patients’ subgroups as well. Even in uncomplicated cases (n = 50), their frequency equalled 66% [*p* < 0.000001, OR = 17.9, 95% CI (8.73–36.55) vs. controls]. We observed no significant differences in the frequency of high (> 2.9 μg/ml) concentrations (AML: 2.6% vs. C: 5.1%). Interestingly, although ficolin-1 levels correlated with white blood cell (WBC) and more weakly with platelet (PLT) counts (r = 0.49, *p* < 0.000001 and r = 0.24, *p* = 0.003, respectively), high leukocytosis was often associated with low ficolin-1. It should be however stressed that no correlation (r = 0.05, *p* = 0.53) with absolute neutrophil count (ANC) was observed. As shown in Fig. 2A, B, serum ficolin-1 concentrations in the AML group were not associated with *FCN1* gene SNP at positions − 542 and − 144 while for the control group, expected trends were apparent (lower levels related to variant alleles), although the differences between carriers of various genotypes were not significant.

### *FCN2* gene polymorphisms and serum concentrations of ficolin-2

We found no significant differences in *FCN2* genotypes or allele distribution between patients and controls (Table 2). Reconstruction of haplotypes revealed *AACG* (including major alleles at positions − 64, − 4, + 6,359 and + 6,424) to be the most common in both AML and C groups. Together with the *AGTG* variant, it accounted for approx. 80% of haplotypes in each group/subgroup (Supplementary Table 2). The *AACG* haplotype was found especially often among patients who had no infective complications during hospital stay (*p* = 0.029 vs. controls). However, that result was not statistically significant after correction for multiple comparisons.

**Table 2:** Frequency of genotypes associated with selected *FCN2* single nucleotide gene polymorphisms.

Polymorphism	Genotype	Group					
		C	AML	AML-A	AML-B	AML-C	AML-D
-64 *A* > *C*	*A/A*	203 (78.7)	126 (80.3)	32 (78)	49 (83.1)	2 (33.3)	43 (84.3)
(rs78654553)	*A/C*	54 (20.9)	29 (18.5)	9 (22)	9 (15.3)	4 (66.7)	7 (13.7)
	*C/C*	1 (0.4)	2 (1.3)	0	1 (1.7)	0	1 (2)
-4 *A* > *G*	*A/A*	105 (40.9)	66 (42)	12 (29.3)	25 (42.4)	4 (66.7)	25 (49)
(rs17514136)	*A/G*	118 (45.9)	74 (47.1)	26 (63.4)	24 (40.7)	2 (33.3)	22 (43.1)
	*G/G*	34 (13.2)	17 (10.8)	3 (7.3)	10 (16.9)	0	4 (7.8)
+ 6,359 *C* > *T*	*C/C*	99 (38.8)	69 (43.9)	12 (29.3)^1^	27 (45.8)	3 (50)	27 (52.9)
(rs175491193)	*C/T*	115 (45.1)	67 (42.7)	24 (58.5)	21 (35.6)	3 (50)	19 (37.3)
	*T/T*	41 (16.1)	21 (13.4)	5 (12.2)	11 (18.6)	0	5 (9.8)
+ 6,424 *G* > *T*	*G/G*	199 (77.4)	129 (82.2)	35 (85.4)	49 (83.1)	2 (33.3)	43 (84.3)
(rs7851696)	*G/T*	58 (22.6)	25 (15.9)	6 (14.6)	9 (15.3)	3 (50)	7 (13.7)
	*T/T*	0	3 (1.9)	0	1 (1.7)	1 (16.7)	1 (2)

Percentages are shown in parentheses. Allele distributions were in accordance with Hardy–Weinberg equlibrium.

C: controls; AML-A: patients who experienced infections with proven bacteremia and/or fungaemia; AML-B: patients who experienced infections with no bacteremia; AML-C: patients who experienced febrile neutropenia; AML-D: patients who experienced none of afore-mentioned complications within 4 weeks of hospital stay.

^1^*p* = 0.033, OR = 0.37, 95% CI (0.15–0.88) (vs. AML-D).

Despite lack of differences between patients and controls in the frequency of *FCN2* genotypes, the median ficolin-2 concentration in the AML group (4.69 µg/ml) was significantly higher than in the controls (3.38 µg/ml, *p* < 0.000001, Mann–Whitney *U*-test). In the specific case of the exon 8 SNP, despite apparent trends, the differences between variant homozygotes (+ 6,359 *C* > *T*) or heterozygotes (+ 6,424 *G* > *T*) were not statistically significant perhaps due to their relatively low numbers (Fig. 2E, F). The highest values were associated with infections whether accompanied by bacteremia/fungaemia (4.69 µg/ml; *p* = 0.00006) or not (5.05 µg/ml; *p* < 0.000001) (Fig. 1B). Unusually, in 10 (6.4%) patients, ficolin-2 level exceeded 10 µg/ml (maximal value 13.6 μg/ml). No significant relationships were found between ficolin-2 concentration and duration of fever or mortality in patients. On the other hand, *C/C* homozygosity for + 6,359 *C* > *T* (rs175491193) polymorphism was less common among patients with confirmed bacteremia/fungaemia (AML-A) compared with those with no hospital infections (AML-D) [*p* = 0.033, OR = 0.37, 95% CI (0.15–0.88), not significant after Bonferroni correction] (Table 2).

Consequently, high ficolin-2 levels (> 6.35 µg/ml) were overrepresented among AML patients (29.5%), in comparison with controls (5.2%) [*p* < 0.00001, OR = 7.65, 95% CI (3.92–14.57); after multiple logistic regression analysis: *p* < 0.001 OR = 5.43 95% CI (2.01–14.63)]. The highest frequency (35.6%) of such high ficolin-2 concentrations was found among patients who experienced infections with no bacteremia/fungaemia [*p* < 0.000001, OR = 9.99, 95% CI (4.62–21.62) vs. C group].

Ficolin-2 levels correlated weakly but significantly with ficolin-1 (r = 0.22, *p* = 0.006) and WBC count (r = 0.22, *p* = 0.009) but not with ANC or PLT counts (not shown). In contrast to ficolin-1, significant (although again relatively weak) correlations with C-reactive protein (CRP) (r = 0.28, *p* = 0.002) and fibrinogen (FBG) (r = 0.29, *p* = 0.003) concentrations were found.

Ficolin-2 concentrations correlated with *FCN2* polymorphisms at positions − 64 and + 6,424 both in patients and in controls although the difference between *G/G* homozygotes and *G/T* heterozygotes in the latter did not reach statistical significance (Fig. 2C, F). However, we did not find the expected associations for the SNPs at positions − 4 and + 6,359 (Fig. 2D, E).

### *FCN3* gene + 1637delC mutation and serum concentrations of ficolin-3

Although only 1 patient out of 157 (0.6%) was heterozygous (*C/delC*), there was no statistically significant difference compared with the controls (7 heterozygotes out of 257 individuals tested, 2.7%). None of individuals tested was variant homozygote (genotype associated with ficolin-3 primary deficiency). As in the case of ficolin-2, the median ficolin-3 serum concentration was markedly higher for AML patients (33.6 μg/ml) compared with controls (19.6 μg/ml, *p* < 0.000001, Mann–Whitney *U*-test). Again, the highest value was associated with infections without bacteremia/fungaemia (36.4 μg/ml, *p* < 0.000001 vs. C group), but significant differences were observed in all subgroups (Fig. 1C). No association of ficolin-3 levels with duration of fever or mortality was evident.

Like ficolin-2, high ficolin-3 concentrations were much more common among patients (42%) than controls (5%) [*p* < 0.000001, OR = 14.1, 95% CI (7.42–26.75); multiple logistic regression analysis and correction for multiple comparisons still showed highly significant result: *p* < 0.001, OR = 17.92 95% CI (6.31–50.93)]. Most (54.2%) patients who experienced infections not associated with bacteremia/fungaemia had high (> 34.9 μg/ml) serum ficolin-3 levels before chemotherapy (*p* < 0.000001, OR = 22.4, 95% CI (10.52–47.83) vs. C group). In contrast, low concentration (< 12.9 μg/ml) was found in one patient only (0.6%) (*FCN3 C/delC* heterozygote) while the frequency of such low ficolin-3 levels among controls reached 10% [*p* < 0.000001, OR = 0.06, 95% CI (0.008–0.43)].

Ficolin-3 weakly but significantly correlated with ficolin-1 (r = 0.17, *p* = 0.038), ficolin-2 (r = 0.31, *p* = 0.0001) and WBC count (r = 0.25, *p* = 0.002), but not with ANC or PLT counts (not shown).

### *MBL2* gene polymorphisms and mannose-binding lectin concentrations

Although the frequency of *MBL2* gene *HYA, LYA, LXA, HYD, LYD, LYB* or *LYC* haplotypes did not differ significantly between the groups (Supplementary Tables 3 and 4), MBL deficiency-associated genotypes (*LXA/O*, *O/O*) were more common in patients suffering from AML than in controls when analysed with the χ^2^ test (*p* = 0.026, OR = 1.9, 95% CI (1.07–3.43). However, after multiple logistic regression, correction for sex and age (recessive model) and Bonferroni correction, no significant difference was found [*p* = 0.093, OR = 1.77, 95% CI (0.91–3.44)] (Table 3). The promoter/exon 1 polymorphisms seemed not to influence risk for hospital infections in patients or duration of fever (not shown).

**Table 3:** Frequency of genotypes associated with selected *MBL2* single nucleotide gene polymorphisms.

Genotype	Group					
	C	AML	AML-A	AML-B	AML-C	AML-D
*A/A*	180 (69.5)	98 (64.1)	29 (74.4)	38 (65.5)	2 (40)	29 (56.9)
*YA/O*	53 (20.5)	28 (18.3)	3 (7.7)	9 (15.5)	2 (40)	14 (27.5)
*XA/O* or *O/O*	26 (10)	27 (17.7)^1^	7 (17.9)	11 (19)	1 (20)	8 (15.7)

Percentages are shown in parentheses. Allele distributions (*H/L, Y/X, A/D, A/B, A/C*) were in accordance with Hardy–Weinberg equlibrium.

C: controls; AML-A: patients who experienced infections with proven bacteremia and/or fungaemia; AML-B: patients who experienced infections with no bacteremia; AML-C: patients who experienced febrile neutropenia; AML-D: patients who experienced none of afore-mentioned complications within 4 weeks of hospital stay.

^1^*p* = 0.026, OR = 1.9, 95% CI (1.07–3.43) [multiple logistic regression, recessive model: *p* = 0.093, OR = 1.77, 95% CI (0.91–3.44)].

The median MBL serum concentration before chemotherapy was significantly higher in AML patients than in controls (1,381 ng/ml vs. 789 ng/ml, *p* = 0.0016, Mann–Whitney *U*-test). That reflected the marked difference between *A/A* homozygotes but not between carriers of one or two *O* alleles. Furthermore, commonly observed, apparent differences in MBL levels between carriers of the same genotypes (especially A/A) were found (Fig. 2G).

The highest median (2,122 ng/ml) was obtained from patients who later had severe infections accompanied with bacteremia/fungaemia (Fig. 1D). It differed significantly from patients who had no short-term infective complications (989 ng/ml, *p* = 0.012). No association of serum MBL level with duration of fever or mortality was found.

Generally, high MBL concentrations (> 3 μg/ml) were overrepresented among patients (26%) compared with controls [4.9%, *p* < 0.000001, OR = 6.7, 95% CI (3.44–12.98); after multiple logistic regression analysis: *p* < 0.001 OR = 9.22 95% CI (3.03–28.02)] with particularly high frequency associated with hospital infections whether accompanied with bacteremia/fungaemia [26.8%, *p* < 0.0001, OR = 7.1, 95% CI (2.93–17.27)] or not [33.9%, *p* < 0.0001, OR = 9.94, 95% CI (4.58–21.59)].

MBL concentration within the AML group inversely correlated with ficolin-1 (r = − 0.18, *p* = 0.02) but not with other ficolins, WBC, ANC, PLT counts, CRP or FBG (not shown).

### Ficolins and mannose-binding lectin as potential supplementary biomarkers of acute myeloid leukaemia

The ROC analysis suggests high potential of ficolin-1 to differentiate between acute myeloid leukaemia patients and healthy subjects. Furthermore, it may well differentiate between persons suffering from AML and multiple myeloma or lymphoma (Table 4; Supplementary Fig. 1). Ficolin-2 had lower potential to differentiate between AML and C groups but more promising data were obtained for multiple myeloma and lymphoma (Table 4; Supplementary Fig. 2). In the case of ficolin-3, the ROC analysis demonstrated relatively high potential to differentiate between AML patients and controls as well as persons suffering from other haematological malignancies (Table 4; Supplementary Fig. 3).

**Table 4:** Ficolins and mannose-binding lectin as potential disease markers (data from ROC analysis).

Protein	AML versus	Curve area	95% CI	Significance	Cut-off (μg/ml)	Sensitivity (%)	Specificity (%)
Ficolin-1	C	0.866	0.825–0.907	*p* < 0.0001	0.55	70.7	94.9
	MM	0.767	0.713–0.821	*p* < 0.0001	0.325	56.7	93.1
	LYMPH	0.695	0.633–0.757	*p* < 0.0001	0.27	51.6	90.5
	MM + LYMPH	0.739	0.685–0.794	*p* < 0.0001	0.27	51.6	94.1
Ficolin-2	C	0.688	0.635–0.742	*p* < 0.0001	5.225	43	83.9
	MM	0.775	0.726–0.825	*p* < 0.0001	3.378	75.6	66.7
	LYMPH	0.838	0.789–0.884	*p* < 0.0001	3.288	78.2	78.3
	MM + LYMPH	0.799	0.756–0.842	*p* < 0.0001	3.288	78.2	69.5
Ficolin-3	C	0.83	0.789–0.871	*p* < 0.0001	24.4	73.9	80.3
	MM	0.806	0.76–0.853	*p* < 0.0001	27.7	68.2	82.2
	LYMPH	0.824	0.776–0.873	*p* < 0.0001	26.4	70.1	82.8
	MM + LYMPH	0.813	0.771–0.856	*p* < 0.0001	27.7	68.2	82.7
MBL	C	0.593	0.532–0.654	*p* = 0.015	1.873	44	81.5
	MM	0.558	0.495–0.62	*p* = 0.065	2.964	27.4	90.9
	LYMPH	0.559	0.491–0.627	*p* = 0.096	2.912	27.4	92.2
	MM + LYMPH	0.558	0.499–0.617	*p* = 0.041	2.964	27.4	91.4

*AML* acute myeloid leukaemia, *C* control (non-malignant), *MM* multiple myeloma, *LYMPH* lymphoma.

Although ROC analysis for MBL revealed statistical significance, the curve area appeared rather low. Furthermore, MBL level has no greater potential to differentiate AML from multiple myeloma or lymphoma (Table 4; Supplementary Fig. 4).

## Discussion

The clinical associations of ficolins with haematological cancers and/or chemotherapy-related infections have not been extensively discussed in the literature. Previously, Schlapbach et al.^25^ reported lower ficolin-1 serum levels in children suffering from AML and acute lymphoblastic leukaemia (ALL) (41 patients in total) than in controls. They found strong positive correlations of ficolin-1 concentration with peripheral blood leukocyte counts and inverse correlations with leukaemic blasts in blood and bone marrow. Our data from a larger cohort of adults diagnosed with AML confirmed a highly significant difference in median ficolin-1 levels between patients and controls (Fig. 1A). As expected, ficolin-1 correlated with WBC and PLT counts; no correlation with neutrophil counts was observed, but that may simply reflect neutropenia including the total lack of neutrophils in 61 out of 143 patients for whom such data were available. In 46 patients, extremely low ficolin-1 (< 100 ng/ml) was found, resulting rather from abnormal haematopoiesis than primary (associated with *FCN1* gene) deficiency. However, the lower median value in the AML group may have a genetic background to some extent, due to higher frequency of the *G/G* genotype (− 542 *G* > *A* SNP) (Table 1). The variant allele (*A*) was previously reported to be associated with higher serum ficolin-1 concentration^19,20^, however we found no significant differences between carriers of different genotypes within both C and AML groups (Fig. 2A, B). The allele distribution among patients is apparently disturbed as it does not comply with Hardy–Weinberg equilibrium (in contrast to controls, as well as to all other SNP studied here, within both groups).

Schlapbach et al.^25^ also reported a lack of association between low ficolin-1 (< 0.5 µg/ml) and febrile neutropenia in paediatric patients (also in those experiencing bacteremia) after chemotherapy. On the other hand, Ameye et al.^26^ found low (median: 270 ng/ml) concentrations of this protein to be associated with severe infections in adults with haematological malignancies (including leukaemias) (median in patients with no such events: 470 ng/ml). Data from our cohort revealed the lowest median for persons with confirmed bacteremia/fungaemia, however low values were also associated with patients with no hospital infections. On the other hand, *A/A* homozygosity related to − 144 *C* > *A* SNP (reported to be associated with higher *FCN1* gene*-*specific mRNA expression and higher serum ficolin-1 level^19,20^) seems to be risk factor for the most severe infections in patients (Table 1). Based on data presented in Supplementary Table 1, it may be speculated that *FCN1 ACG* and *GAG* haplotypes (reconstructed with the EM maximum-likelihood method) are risk factors for acute myeloid leukaemia, especially complicated with severe hospital infections while *GCG* seems to be protective.

The ROC analysis demonstrated high potential of ficolin-1 to discriminate not only between AML patients and healthy controls but also between AML patients and individuals suffering from other haematological malignancies [multiple myeloma or lymphomas; detailed data concerning serum concentrations of ficolins in those patients were recently published^27^ (Table 4). Such a significant association was not reported earlier, although ficolin-1 was suggested to be candidate for marker of other cancers: colorectal cancer and adenoma^28^. Our assumption may be supported by data published by Handschuh et al.^29^ who demonstrated marked underexpression of the *FCN1* gene in peripheral blood mononuclear cells and bone marrow from patients diagnosed with AML. However, Rasmussen et al.^30^, based on analysis concerning a heterogeneous group of cancer patients, suggested ficolin-1 to be useless as disease biomarker.

In contrast to ficolin-1, the median ficolin-2 and ficolin-3 serum concentrations in the AML group were markedly higher than in healthy controls (Fig. 1B, C). In some cases, we observed unusually high ficolin-2 concentrations. Although the highest medians and highest frequencies of high values were generally noted in patients with hospital infections, we found no associations with duration of fever or mortality. It may be assumed that high levels of ficolin-2 and ficolin-3 are associated rather with cancer itself than with chemotherapy-related hospital infections. Ficolin-2, however, unlike ficolin-1 and -3, correlated with the inflammatory markers, CRP and FBG.

Several earlier reports^31,32^ suggested a lack of association of ficolin-2 and/or ficolin-3 with incidence of infections or febrile neutropenia in patients with various haematological cancers. However, low ficolin-2 was suggested to predict higher risk of development of sinusoidal obstruction syndrome (SOS) after allogeneic haemopoietic cell transplantation^33,34^ while low ficolin-3 was considered a risk factor for febrile neutropenia (also accompanied with bacteremia) in children, treated with anti-cancer chemotherapy^35^.

Pana et al.^36^ found associations of certain *FCN2* haplotypes [corresponding to promoter polymorphisms at positions: − 986 (*A* > *G*), − 602 (*G* > *A*), − 4 (*A* > *G*) and exon 8 polymorphisms at positions: + 6,359 (*C* > *T*), + 6,424 (*G* > *T*)] with bacterial infections and duration of febrile neutropenia in children diagnosed with B-cell ALL, after chemotherapy. We have investigated 3 of 5 afore-mentioned polymorphic sites, and one additional (− 64 *A* > *C*). None of them was associated with AML itself (nor were reconstructed haplotypes, demonstrated in Supplementary Table 2), however, major (*C*) allele homozygosity related to + 6,359 *C* > *T* SNP might be considered protective from infections accompanied with bacteremia/fungaemia (Table 2).

The ROC analysis showed high potential of both ficolin-2 and ficolin-3 to differentiate between AML patients and controls as well as patients suffering from multiple myeloma or lymphomas (Table 4). Earlier, Rasmussen et al.^30^ found no discriminating potency of ficolin-3 between cancer patients in general and healthy individuals or persons with non-malignant diseases while Storm et al.^28^ reported its rather weak potential to differentiate between colorectal cancer and adenoma patients (but not controls). In both afore-mentioned investigations, ficolin-2 concentrations were not determined.

Data from single *MBL2* gene analysis suggested that primary MBL deficiency (*MBL2 LXA/O* or *O/O* genotypes) may be associated with a higher risk to develop AML, however it was not confirmed in multiple logistic regression analysis and correction for multiple comparisons (*p* > 0.05). It furthermore did not influence risk for chemotherapy-related infections in patients. Recently we found an association between inherited MBL deficiency and multiple myeloma but not hospital infections after autologous haematopoietic stem cell transplantations^37^. Earlier, Schmiegielow et al.^38^ reported MBL-deficient genotypes to be associated with another type of leukaemia (ALL) in children. The lack of active MBL molecules may therefore be involved in development of both AML and ALL, in spite of differences in their aetiopathogenesis. Despite the possible association of MBL deficiency with cancer, we generally observed higher serum MBL in patients. Interestingly, earlier we reported similar findings in the context of ovarian cancer: MBL deficiency was associated with the disease itself but in parallel, MBL serum levels in patients (before surgery) were significantly higher than in controls which reflected mainly the difference between *A/A* homozygous women^17^, as also we report here for persons suffering from AML (Fig. 2G). Furthermore, high MBL in AML patients seemed to predict the most severe infections (accompanied with bacteremia and/or fungaemia). This may support our previous hypothesis^37^ that since during cytopenia, the opsonic effect of MBL does not promote phagocytosis, even a high level is not protective against infections despite the ability to activate complement. Another, rather speculative explanation, may be presence of asymptomatic/latent infections resulting in elevated MBL and being (re)activated when immunosuppression is escalated by chemotherapy. Similarly, the lack of association of mannose-binding lectin deficiency with chemotherapy-induced infections in patients suffering from acute myeloid leukaemia was earlier reported by Bergmann et al.^39^ and Klostergaard et al.^40^. Furthermore, data from several other studies (concerning patients with other leukaemias, or haematological malignancies in general) led to similar conclusions^41–46^. In contrast to our results presented here and in afore-mentioned recent paper^37^, some earlier reports suggested that low MBL (or associated genotypes) enhanced the risk for infections in such cases^31,32,36,47–50^. Furthermore, it was demonstrated that L-asparaginase (used in ALL treatment) induces a marked decrease in MBL concentration, which was suspected to contribute to increased risk of febrile neutropenia with infection^51^. Therefore, involvement of MBL in haematological malignancies appears complex and equivocal. It seems to depend on variety of factors, including patients genotype, age, type of disease, treatment regimen, etc.

Despite significant difference in MBL serum concentrations between patients and controls, this lectin does not seem to be a promising candidate for biomarker of acute myeloid leukaemia (Fig. 1D, Table 4). Earlier, Storm et al.^28^ and Rasmussen et al.^30^ found no discriminating potency of mannose-binding lectin between cancer patients and healthy individuals.

Generally, the significant differences in serum concentrations of ficolins and MBL between patients and controls seem to reflect dysregulation of expression of corresponding genes. This is most evident for ficolin-1 in the light of its major sites of synthesis (bone marrow and leukocytes), its known underexpression in AML^29^ as well as data presented in Fig. 2A, B. Other genes analysed here (*FCN2, FCN3, MBL2*) are primarily expressed in the liver and their products present in blood are mainly of hepatic origin. As liver involvement with AML is uncommon^52^, it may be supposed that their overexpression depends on a changed profile of factors regulating their synthesis associated with abnormal haematopoiesis and carcinogenesis. For example, serum level of interleukin-6 (IL-6) which is known to increase expression of MBL^53^ was shown to be higher in AML patients than in healthy controls^54^. Similarly, expression of *FCN2* and *FCN3* genes in the liver may be affected by higher concentrations of pro-inflammatory agents or lower levels of suppressing factors.

Another problem is the potential involvement of abnormal expression of ficolins in organs/tissues other than liver, especially ficolin-3 in the respiratory system. Ficolin-3 seems not to protect from local infections as pneumonia (often severe) is common among AML patients. The normally low expression of the *FCN3* and *FCN2* genes in adrenal glands and adipose tissue as well as *FCN3* in kidney, and *FCN2* in prostate (reviewed by Garred^55^) might also be affected by disease, however there is no data published concerning their associated adverse effects. Dysregulated expression of the *FCN2* gene (like the afore-mentioned *FCN1*) might also explain the unexpected lack of difference in ficolin-2 serum levels, depending on − 4 *A* > *G* and + 6,359 *C* > *T* SNP (Fig. 2D, E). However, similar results were noted in healthy controls, therefore such a direct association is rather unlikely.

Some limitations of our study, such as possible influence of the type of blood collection tubes or sample handling procedures on ficolin-1 and ficolin-2 levels^56–58^ or correlation of ficolin-3 with age^59^^,^ have to be taken into account but they may be simply overcome. Another limitation may be the relatively small sample size and certain heterogeneity of the AML group (some patients had relapsed). However, most of the associations (especially those concerning serum levels of ficolins) are highly significant and no greater differences in concentrations were observed between newly diagnosed and relapsed patients (not shown).

**Figure 3:**
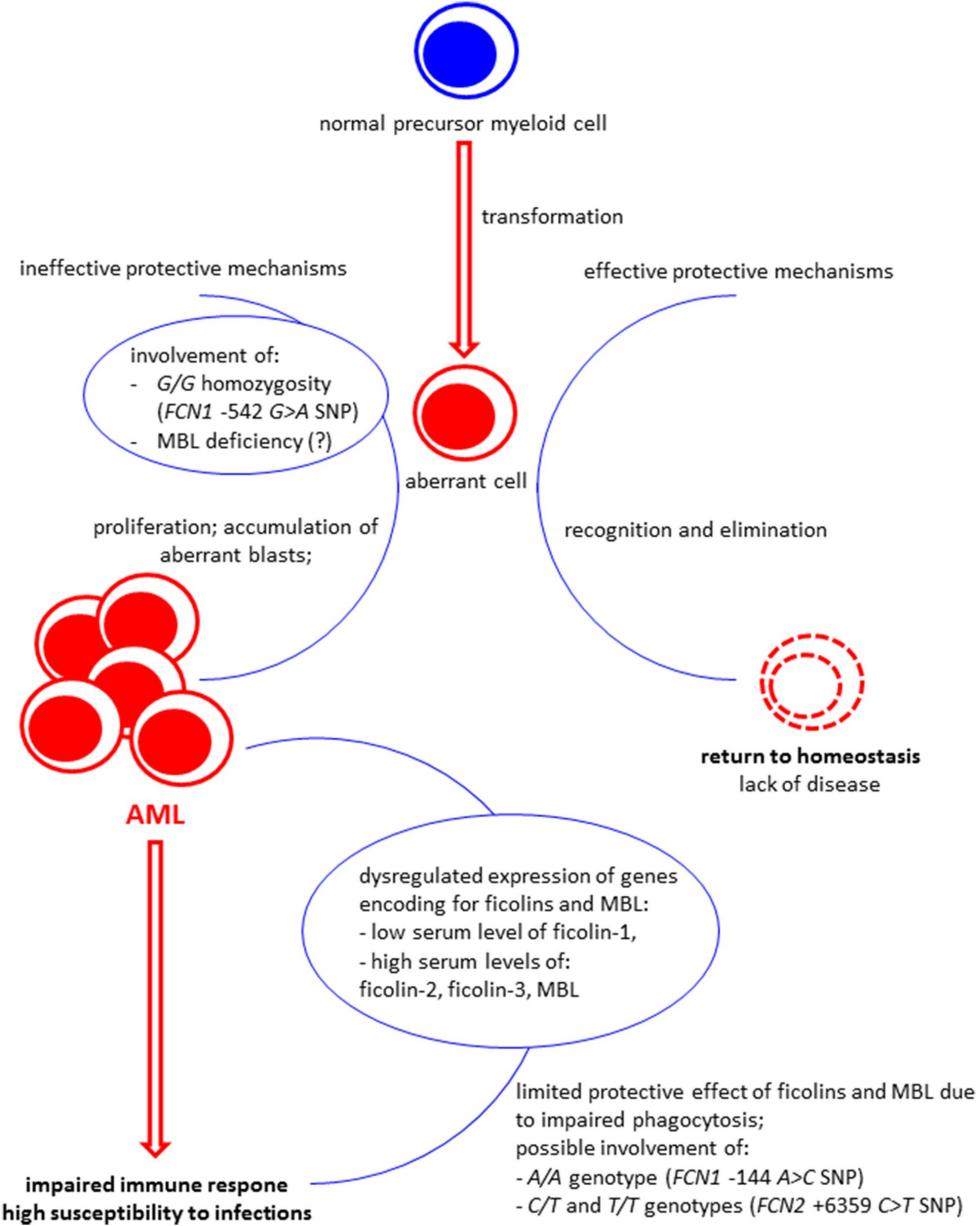
Scheme presenting hypothetical explanation of our results. The G/G homozygosity for the *FCN1* gene − 542 *G* > *A* polymorphism (related to relatively low ficolin-1 gene expression) and possibly primary MBL deficiency may contribute to impaired clearance of aberrant cells and thus to disease development/progression. That leads to dysregulation of expression of genes encoding for ficolins and MBL, resulting in low ficolin-1 and high ficolin-2, ficolin-3 and MBL (in MBL-sufficient patients) serum concentrations. Aberrant haematopoiesis is associated with impaired immune response to pathogens which is further affected by afore-mentioned dysregulation of production of soluble pattern recognition molecules and chemotherapy (not shown in figure). The protective effect of ficolins and MBL seems to be limited due to impaired phagocytosis (even ficolin- or MBL-opsonized bacterial/fungal cells cannot be eliminated by phagocytes). However, *A/A* (− 144 *C* > *A, FCN1* gene), *C/T* and *T/T* (+ 6,359 *C* > *T, FCN2* gene) genotypes were found to be associated with the most severe infections within short period after chemotherapy).

To summarize, our data suggest that ficolins may be considered candidate supplementary biomarkers of AML: low ficolin-1 as well as high ficolin-2 and ficolin-3 seem to be associated with that disease and differentiate patients not only from healthy controls but also from persons suffering from some other haematological malignancies (at least two groups of cancers: multiple myeloma and lymphoma). However, the most important findings reported here are possible associations of *FCN1* and probably to less extent *MBL2* gene polymorphisms with malignancy. An impact of serum MBL on risk of severe infections may be of clinical significance. The hypothetical explanation of our data is presented in Fig. 3.

## Material and methods

### Patients and controls

One hundred and fifty seven patients suffering from acute myeloid leukaemia (134 newly diagnosed; 23 relapsed) were recruited (73 females and 84 males, mean age: 60.3 ± 14.9; age range 24–89). Eighty-one persons were treated according to the 3 + 7 scheme [(cytarabine, administered at days 1–7 with anthracycline (daunorubicin or idarubicin, given at days1-3); dosages depending on patient’s age] or DAC scheme (daunorubicine, cytarabine with the addition of cladribine, for 1–5 days), as recommended by Polish Acute Leukemia Group (PALG)^60^. The remaining patients were treated with cytarabine alone (n = 18), azicitidine (n = 14), decitabine (n = 11) or other chemotherapeutic agents (n = 17). Sixteen patients not considered for any chemotherapy underwent best supportive care (BSC) only. Crucial clinical parameters like white blood cell (WBC) count, absolute neutrophil count (ANC), platelet (PLT) count, inflammatory markers [C-reactive protein (CRP) and fibrinogen (FBG)] levels were determined. During hospital stay, incidence of complications [infections (associated with bacteremia/fungaemia or not), febrile neutropenia (FN); duration of fever > 38 °C without clinical symptoms of infection in neutropenic patients] were recorded and used for analyses for 4 weeks. Forty patients died within a short period (up to 1 month) after starting chemotherapy. Furthermore, 23 deaths were noted among 96 persons followed-up for at least 6 months. Hospital infections were confirmed in 100 patients (in 41 persons accompanied with bacteremia/fungemia, defined as severe infections), in six cases febrile neutropenia with no diagnosed infection was observed. The most common aetiological agents were staphylococci (26 isolates, including 8 methicilin-resistant, MR) and *E. coli* (13 isolates, including 6 extended spectrum betalactamases-positive, ESBL+).

The control group included 267 individuals (unrelated volunteers with no history of cancer, autoimmune diseases or recurrent infections; 174 females and 93 males; mean age: 48 ± 13; age range 18–84). More detailed characteristics of the control group, was previously published by Świerzko et al.^37^. Additionally, for some analyses, data from patients suffering from other haematological malignancies: multiple myeloma (n = 194) or lymphomas (n = 118) were used (those groups were described in detail previously^37^).

The study was approved by the Ethics Committee of the Medical University of Łódź and *w*ritten informed consent from patients and controls was obtained. This work conforms to the provisions of the Declaration of Helsinki.

### DNA and serum samples

Blood samples for DNA extraction were taken from patients into S-Monovette citrated tubes (Sarstedt, Nümbrecht, Germany) before chemotherapy and stored at − 80 °C. DNA was extracted with the use of GeneMATRIX Quick Blood Purification Kit (EURx Ltd, Gdańsk, Poland), according to the manufacturer’s protocol. Samples for serum isolation were taken into S-Monovette tubes with clot activator (Sarstedt) immediately before starting chemotherapy. Sera were stored at − 80 °C until testing.

### *FCN1* genotyping

Polymorphisms at positions + 6,658 (*G* > *A*; A218T; exon 8, rs148649884) and + 7,895 (*T* > *C*; S268P; exon 9, rs150625869) were investigated using PCR–RFLP procedures, with the help of HpyCH4V (New England Biolabs, Ipswich, MA, USA) and Taq1 (Thermo Fisher Scientific, Waltham, MA, USA) endonucleases, respectively, essentially as described by Świerzko et al.^61^.

Another SNP, located in exon 9 (+ 7,959 *A* > *G*; N299S; rs138055828) and two located in the promoter region (− 542 *G* > *A*, rs10120023 and − 144 *C* > *A*, rs10117466) were tested with PCR–RFLP as well, using in-house procedures^27^. Briefly, PCRs were run on a C1000 Thermal Cycler (Bio-Rad, Hercules, CA, USA), using appropriate spanning primers, designed with the use of PRIMER3 software (version 0.3.0), https://bioinfo.ut.ee/primer3/^62,63^ (Table 5). DNA samples (100 ng) were added to a reaction volume of 25 μl, containing 2.5 μl of 10 × polymerase buffer, 1 U of TaqDNA polymerase (Thermo Fisher Scientific), 2 mM of MgCl_2_, 200 μM of dNTP mix (Thermo Fisher Scientific) and 0.4 μM of primers.

**Table 5:** Sequences of primers, lenghts of PCR products/restriction fragments and corresponding restriction enzymes, used for PCR–RFLP procedures for investigation of selected *FCN1* gene single nucleotide polymorphisms.

SNP	Primers (forward/reverse)	PCR product (bp)	Enzyme	Restriction fragments (bp)
-542 *G* > *A*	5′-CCCAGAAAATTCAGGGTTTG-3′	148	Taq1	123 + 25
rs10120023	5′-TAACTTTCAAATAATTTACTCCATC-3′		65 °C, 24 h	
-144 *C* > *A*	5′-TGAAGAGTCCCCCAGCTCT-3′	150	BsuRI (HaeIII)	130 + 20
rs10117466	5′-GGAAACATCCTTTGAGATGGC-3′		37 °C, 24 h	
+ 7,959 *A* > *G*	5′-CACTAGCAGGTGCATGTGGA-3′	177	Tru1I	156 + 21
rs138055828	5′-CGACTGTCATGCTTCAAACCTTA-3′		65 °C, 24 h	

The PCR conditions were as follows:

95 °C for 3 min; 59 °C for 30 s, then 35 cycles (72 °C for 30 s, 95 °C for 30 s, 59 °C for 30 s) plus final elongation 72 °C for 3 min. PCR products were treated with restriction enzymes [all coming from Thermo Fisher Scientific, in buffers recommended by the producer; conditions given in Table 5]. Products corresponding to alleles *G* (position − 542), *C* (− 144) and *A* (+ 7,959) underwent digestion while those corresponding to alleles *A*, *A*, *G,* respectively remained intact. The lengths of restriction fragments are presented in Table 5. In preliminary experiments, genotypes have been confirmed with the help of sequencing (not shown).

### *FCN2* genotyping

Polymorphisms of the *FCN2* gene: − 64 *A* > *C* (rs78654553), (located within promoter), + 6,359 *C* > *T* (rs175491193), and + 6,424 *G* > *T* (rs7851696) (both exon 8) were analysed using allele-specific PCR, as described previously^64^. Another promoter polymorphism, − 4 *A* > *G* (rs17514136) was investigated using PCR–RFLP procedure, employing MboII restriction enzyme (Thermo Fisher Scientific)^64^.

### *FCN3* genotyping

The presence of + 1637*delC* frameshift mutation of the *FCN3* gene was investigated using PCR–RFLP procedure, with the help of ApaI endonuclease (Thermo Fisher Scientific), as previously described by Michalski et al.^65^.

### *MBL2* genotyping

Single nucleotide polymorphisms of the *MBL2* gene, localised to promoter (*H/L*, at position − 550, rs11003125 and *Y/X*, at position − 221, rs7096206) were analysed using allele-specific PCR, as described previously^66^. Exon 1 (*A/D*, codon 52, rs5030737; *A/B*, codon 54, rs1800450 and *A/C*, codon 57, rs1800451) polymorphisms were investigated with the use of PCR–RFLP procedures, employing MluI, BshNI and MboII (all purchased from Thermo Fisher Scientific) enzymes, respectively^66^.

### Determination of serum concentrations of ficolins and mannose-binding lectin

Serum ficolin-1 and ficolin-3 concentrations were determined by TRIFMA (as described by Wittenborn et al.^67^) and ELISA [according to Michalski et al.^65^), respectively. Ficolin-2 levels were measured by TRIFMA. Briefly, microtitre plates (Optiplate-384HB, Perkin Elmer, Waltham, MA, USA) were coated with anti-ficolin-2 (ABS 005-16, BioPorto Diagnostics, Copenhagen, Denmark, 1 µg/ml). Plates were blocked with 0.1% BSA and then incubated with sera to be tested, pre-diluted 1:10. Biotinylated mAb (clone GN4, 1:100, Hycult Biotech, Uden, The Netherlands) and Eu^3+^-labeled streptavidin (Perkin Elmer) were used for detection. After incubation with enhancement solution (Perkin Elmer), fluorescence values were measured using Varioskan Flash reader (Thermo Fisher Scientific, Waltham, MA, USA). Serum from a healthy volunteer (ficolin-2 concentration: 3.5 μg/ml) was used as a standard. MBL concentrations were determined by ELISA, using mannan from *Saccharomyces cerevisiae* (Sigma-Aldrich, St. Louis, MO, USA) as coating antigen and murine anti-human MBL mAbs (HYB131-1, BioPorto Diagnostics, Copenhagen, Denmark)^68^.

“Low” and “high” values were arbitrarily based on 10th and 95th percentiles respectively, determined for the control group. Consequently, concentrations < 620 ng/ml (ficolin-1), < 1,670 ng/ml (ficolin-2) and < 12.9 µg/ml (ficolin-3) were considered “low” while > 2.9 µg/ml, > 6.35 µg/ml and > 34.9 µg/ml, respectively were considered “high”^27^. Exceptionally, MBL was considered “low” when its concentration was < 150 ng/ml (as MBL deficiency) while “high” MBL consequently corresponded to the 95th percentile for the control group (> 3 µg/ml)^37^.

### Statistical analysis

The Statistica (version 13.3, TIBCO Software), SigmaPlot (version 12.0, Systat Software) and Arlequin (version 3.5.2.2, https://cmpg.unibe.ch/software/arlequin35/) software packages were used for data management and statistical calculations. The medians of protein concentrations were compared using the Mann–Whitney *U*-test. It was chosen because values were not normally distributed (not shown). The frequencies of low or high levels, as well as genotypes/alleles were compared by two-sided Fischer’s exact test (or χ^2^ when appropriate). The EM maximum-likelihood method was used for reconstruction of *FCN1* and *FCN2* haplotypes. Possible associations were verified with multiple logistic regression analysis, corrected for sex and age. Furthermore, analyses were corrected for multiple comparisons (Bonferroni correction). Two SNP (*FCN1* gene: + 7,895 T > *C,* + 7,959 *A* > *G*) were excluded from analysis (no heterozygotes or variant homozygotes found). Furthermore, another SNP of the same gene (+ 6,658 *G* > *A*) and *FCN3* gene + 1637 *C* > *delC* mutation were excluded from recessive model (no variant homozygotes). For estimation of differentiating potential of tested factors, ROC analysis was employed. Correlations were determined by Spearman’s test. *p* values < 0.05 (*p* < 0.0125 after correction for multiple comparisons) were considered statistically significant.

## Supplementary information

Supplementary information

## Abbreviations

AML: Acute myeloid leukaemia
ANC: Absolute neutrophil count
FN: Febrile neutropenia
MBL: Mannose-binding lectin
PLT: Platelet count
WBC: Leukocyte count

## References

[1] Wierzbowska A, Czemerska M (2013). Acute myeloid leukemia in the elderly people. Acta Haematol. Pol..

[2] Austin R, Smyth MJ, Lane SW (2016). Harnessing the immune system in acute myeloid leukaemia. Crit. Rev. Oncol. Hematol..

[3] Prada-Arismendy J, Arroyave JC, Rothlisberger S (2017). Molecular biomarkers in acute myeloid leukemia. Blood Rev..

[4] Short NJ, Rytting ME, Cortes JE (2018). Acute myeloid leukaemia. Lancet.

[5] Shallis RM, Wang R, Davidoff A, Ma X, Zeidan AM (2019). Epidemiology of acute myeloid leukemia: recent progress and enduring challenges. Blood Rev..

[6] Khayr W, Haddad RY, Noor SA (2012). Infections in hematological malignancies. Dis. Mon..

[7] Ruhnke M, Arnold R, Gastmeier P (2014). Infection control issues in patients with haematological malignancies in the era of multidrug-resistant bacteria. Lancet Oncol..

[8] Gedik H (2014). Bloodstream infections in patients with hematological malignancies: which is more fatal—cancer or resistant pathogens?. Ther. Clin. Risk Manag..

[9] Nucci M, Anaissie EJ, Wiernik P, Dutcher J, Gertz M (2018). Prevention of infections in patients with hematological malignancies. Neoplastic Diseases of the Blood.

[10] Cedzyński M, Kilpatrick DC, Świerzko AS, Barnum S, Schein T (2018). Mannose-binding lectin. The Complement Factsbook.

[11] Matsushita M, Barnum S, Schein T (2018). Ficolins. The Complement Factsbook.

[12] Nakagawa T, Kawasaki N, Ma Y, Uemura K, Kawasaki T (2003). Antitumor activity of mannose-binding protein. Methods Enzymol..

[13] Yang G (2016). FCN2 inhibits epithelial-mesenchymal transition-induced metastasis of hepatocellular carcinoma via TGF-β/Smad signaling. Cancer Lett..

[14] Ding Q (2017). Ficolin-2 triggers antitumor effect by activating macrophages and CD8^+^ T cells. Clin. Immunol..

[15] Michalski M (2019). Interactions of ficolin-3 with ovarian cancer cells. Immunobiology.

[16] Pągowska-Klimek I (2016). Activation of the lectin pathway of complement by cardiopulmonary bypass contributes to the development of systemic inflammatory response syndrome after paediatric cardiac surgery. Clin. Exp. Immunol..

[17] Swierzko AS (2014). Mannose-Binding Lectin (MBL) and MBL-associated serine protease-2 (MASP-2) in women with malignant and benign ovarian tumours. Cancer Immunol. Immunother..

[18] Michalski M (2019). Factors involved in initiation and regulation of complement lectin pathway influence postoperative outcome after pediatric cardiac surgery involving cardiopulmonary bypass. Sci. Rep..

[19] Ammitzboll CG (2012). Non-synonymous polymorphisms in the *FCN1* gene determine ligand-binding ability and serum levels of M-ficolin. PLoS ONE.

[20] Munthe-Fog L (2012). Variation in *FCN1* affects biosynthesis of ficolin-1 and is associated with outcome of systemic inflammation. Genes Immun..

[21] Hummelshoj T, Munthe-Fog L, Madsen HO, Fujita T, Matsushita M, Garred P (2005). Polymorphisms in the *FCN2* gene determine serum variation and function of Ficolin-2. Hum. Mol. Genet..

[22] Munthe-Fog L (2007). The impact of *FCN2* polymorphisms and haplotypes on the ficolin-2 serum levels. Scand. J. Immunol..

[23] Cedzynski M (2007). Extremes of L-ficolin concentration in children with recurrent infections are associated with single nucleotide polymorphisms in the *FCN2* gene. Clin. Exp. Immunol..

[24] Munthe-Fog L, Hummelshoj T, Honore C, Madsen HO, Permin H, Garred P (2009). Immunodeficiency associated with *FCN3* mutation and ficolin-3 deficiency. N. Engl. J. Med..

[25] Schlapbach LJ (2011). M-ficolin in children with cancer. Immunobiology.

[26] Ameye L, Paesmans M, Thiel S, Jensenius JC, Aoun M (2012). M-ficolin levels are associated with the occurrence of severe infections in patients with haematological cancer undergoing chemotherapy. Clin. Exp. Immunol..

[27] Świerzko AS (2020). Associations of ficolins with haematological malignancies in patients receiving high-dose chemotherapy and autologous haematopoietic stem cell transplantations (auto-HSCT). Front. Immunol..

[28] Storm L, Christensen IJ, Jensenius JC, Nielsen HJ, Thiel S, Danish Study Group on Early Detection of Colorectal Cancer (2015). Evaluation of complement proteins as screening markers for colorectal cancer. Cancer Immunol. Immunother..

[29] Handschuh L (2018). Gene expression profiling of acute myeloid leukemia samples from adult patients with AML-M1 and -M2 through boutique microarrays, real-time PCR and droplet digital PCR. Int. J. Oncol..

[30] Rasmussen LJH (2017). Inflammatory biomarkers and cancer: CRP and suPAR as markers of incident cancer in patients with serious nonspecific symptoms and signs of cancer. Int. J. Cancer..

[31] Kilpatrick DC (2003). No strong relationship between mannan binding lectin or plasma ficolins and chemotherapy-related infections. Clin. Exp. Immunol..

[32] Islak Mutcali S (2016). Early changes of mannose-binding lectin, H-ficolin, and procalcitonin in patients with febrile neutropenia: a prospective observational study. Turk. J. Hematol..

[33] Akil A (2015). Biomarkers for diagnosis and prognosis of sinusoidal obstruction syndrome after hematopoietic cell transplantation. Biol. Blood Marrow Transplant..

[34] Abu Zaid M (2017). Plasma biomarkers of risk for death in a multicentre phase 3 trial with uniform transplant characteristics post-allogenic HCT. Blood.

[35] Schlapbach LJ (2009). H-ficolin serum concentration and susceptibility to fever and neutropenia in paediatric cancer patients. Clin. Exp. Immunol..

[36] Pana ZD (2014). Mannose-binding lectin and ficolin-2 polymorphisms are associated with increased risk for bacterial infections in children with B acute lymphoblastic leukemia. Pediatr. Blood Cancer.

[37] Świerzko AS (2018). The role of complement activating collectins and associated serine proteases in patients with hematological malignancies, receiving high-dose chemotherapy, and autologous hematopoietic stem cell transplantations (auto-HSCT). Front. Immunol.

[38] Schmiegielow K (2002). Increased frequency of mannose-binding lectin insufficiency among children with acute lymphoblastic leukaemia. Blood.

[39] Bergmann OJ (2003). Low levels of mannose-binding lectin do not affect occurrence of severe infections or duration of fever in acute myeloid leukaemia during remission induction therapy. Eur. J. Haematol..

[40] Klostergaard A (2010). Sepsis in acute myeloid leukaemia patients receiving high-dose chemotherapy: no impact of chitotriosidase and mannose-binding lectin polymorphisms. Eur. J. Haematol..

[41] Choi EH (2005). Common polymorphisms in critical genes of innate immunity do not contribute to the risk for chronic disseminated candidiasis in adult leukemia patients. Med. Mycol..

[42] Lausen B, Schmiegielow K, Andersen B, Madsen HO, Garred P (2006). Infections during induction therapy of childhood acute lymphoblastic leukemia – no association to mannose-binding lectin deficiency. Eur. J. Haematol..

[43] Frakking FJN (2011). Mannose-binding lectin (MBL) and the risk for febrile neutropenia and infection in pediatric oncology patients with chemotherapy. Pediatr. Blood Cancer.

[44] Te Poele EM (2011). *MBL2* and fever during neutropenia in children with acute lymphoblastic leukaemia. Br. J. Haematol..

[45] Wong M (2012). Mannose-binding lectin 2 polymorphisms do not influence frequency or type of infection in adults with chemotherapy induced neutropaenia. PLoS ONE.

[46] Holanda K (2014). Mannose-binding lectin 2 (*MBL2*) gene polymorphisms do not influence frequency of Infections in chronic lymphocytic leukemia patients. Rev. Bras. Hematol. Hemoter..

[47] Peterslund NA, Koch C, Jensenius JC, Thiel S (2001). Association between deficiency of mannose-binding lectin and severe infections after chemotherapy. Lancet.

[48] Vekemans M (2007). Low mannose-binding lectin concentration is associated with severe infections in patients with haematological cancer who are undergoing chemotherapy. Clin. Infect. Dis..

[49] Ghazi M (2012). Serum levels of mannose-binding lectin and the risk of infection in pediatric oncology patients with chemotherapy. J. Pediatr. Hematol. Oncol..

[50] Dommet R, Chisholm J, Turner M, Bajaj-Elliott M, Klein NJ (2013). Mannose-binding lectin genotype influences frequency and duration of infectious complications in children with malignancy. J. Pediatr. Hematol. Oncol..

[51] Merlen C (2015). L-asparaginase lowers plasma antithrombin and mannan-binding lectin levels: impact of thrombotic and infectious events in children with acute lymphoblastic leukemia. Pediatr. Blood Cancer.

[52] Mathews E, Laurie T, O’Riordan K, Nabhan C (2008). Liver involvement in acute myeloid leukemia. Case Rep. Gastroenterol..

[53] Sorensen CE, Hansen TK, Steffensen R, Jensenius JC, Thiel S (2006). Hormonal regulation of mannan-binding lectin synthesis in hepatocytes. Clin. Exp. Immunol..

[54] Ahmed HS, Tahir NT, Obed FA (2017). Cytokines profiling as prognostic markers in newly diagnosed acute myeloid leukemia. Iraqi J. Hematol..

[55] Garred P (2016). A journey through the lectin pathway of complement - MBL and beyond. Immunol. Rev..

[56] Hein E, Bay JT, Munthe-Fog L, Garred P (2013). Ficolin-2 reveals different analytical and biological properties dependent on different sample handling procedures. Mol. Immunol..

[57] Brady AM, Spencer BL, Falsey AR, Nahm MH (2014). Blood collection tubes influence serum ficolin-1 and ficolin-2 levels. Clin. Vaccine Immunol..

[58] Geno KA, Kennedy RE, Sawyer P, Brown CJ, Nahm MH (2016). Ficolin-2 inhibitors are present in sera after prolonged storage at − 80 °C. Peer J..

[59] Troldborg A (2016). Lectin complement pathway proteins in healthy individuals. Clin. Exp. Immunol..

[60] Wierzbowska A (2015). Advances in the treatment of adult patients with acute leukemias. Hematologia.

[61] Świerzko AS (2016). Components of the lectin pathway of complement activation in paediatric patients of intensive care units. Immunobiology.

[62] Untergasser A (2012). Primer3—new capabilities and interfaces. Nucleic Acids Res..

[63] Koressaar T, Remm M (2007). Enhancements and modifications of primer design program Primer3. Bioinformatics.

[64] Szala A, Swierzko AS, Cedzynski M (2013). Cost-effective procedures for genotyping of human *FCN2* gene single nucleotide polymorphisms. Immunogenetics.

[65] Michalski M (2012). H-ficolin (ficolin-3) concentrations and *FCN3* gene polymorphism in neonates. Immunobiology.

[66] Bak-Romaniszyn L (2011). Mannan-binding lectin deficiency in pediatric patients with inflammatory bowel disease. Scand. J. Gastroenterol..

[67] Wittenborn T, Thiel S, Jensen L, Nielsen HJ, Jensenius JC (2010). Characteristics and biological variations of M-ficolin, a pattern recognition molecule, in plasma. J. Innate Immun..

[68] Cedzynski M (2004). Mannan-binding lectin insufficiency in children with recurrent infections of the respiratory system. Clin. Exp. Immunol..

