# Supplementary material for: Associations of ficolins and mannose-binding lectin with acute myeloid leukaemia in adults
Source: Sci Rep. 2020 Jun 29;10:10561. doi: 10.1038/s41598-020-67516-2 (PMC7324623; doi:10.1038/s41598-020-67516-2)
Supplement: Supplementary file 1 — Supplementary information [file 41598_2020_67516_MOESM1_ESM.pdf]

Associations of ficolins and mannose-binding lectin with acute myeloid leukaemia in adults

Anna Sokołowska[1]. Anna S. Świerzko[1]. Gabriela Gajek[1]. Aleksandra Gołos[2]. Mateusz Michalski[1]. Mateusz Nowicki[3]. Agnieszka Szala-Poździej[1]. Anna Wolska-Washer[4]. Olga Brzezińska[5.6]. Agnieszka Wierzbowska[4]. Krzysztof Jamroziak[2]. Marek L. Kowalski[5]. Steffen Thiel[7]. Misao Matsushita[8]. Jens C. Jensenius[7]. Maciej Cedzyński[1]*

[1]Laboratory of Immunobiology of Infections. Institute of Medical Biology. Polish Academy of Sciences. Lodowa 106. 93-232 Łódź. Poland; [2]Department of Hematology. Institute of Hematology and Transfusion Medicine, I. Gandhi 14. 02-776. Warsaw. Poland; [3]Department of Hematology. Copernicus Memorial Hospital in Łódź Comprehensive Cancer Center and Traumatology. Pabianicka 62. 93-513 Łódź. Poland ; [4]Department of Hematology. Medical University of Łódź. Ciołkowskiego 2. 93-510 Łódź. Poland; [5]Department of Immunology and Allergy. Medical University of Łódź. Pomorska 251. 92-213 Łódź. Poland; [6]Department of Rheumatology. Medical University of Łódź. Pieniny 30. 92-003 Łódź. Poland; [7]Department of Biomedicine. Aarhus University. Høegh-Guldbergs Gade 10. 8000 Aarhus C. Denmark; [8]Department of Applied Biochemistry. Tokai University. 4-1-1 Kitakaname. Hiratsuka. Kanagawa 259-1292. Japan

* - corresponding author (mcedzynski@cbm.pan.pl)

Supplementary Table 1. Estimated frequency of reconstructed *FCN1* haplotypes (with EM maximum likelihood method) in patients and controls. C: controls; AML-A: patients who experienced infections with proven bacteremia and/or fungaemia; AML-B: patients who experienced infections with no bacteremia; AML-C: patients who experienced febrile neutropenia; AML-D: patients who experienced none of afore-mentioned complications within 4 weeks of hospital stay. Single nucleotide polymorphisms: -542 *G>A*, -144 *C>A* and +6658 *G>A* were used for reconstruction. *P* values are given when <0.05; results significant after correction for multiple comparisons are displayed in bold.

**Haplotype**	**Group**					
	**C**	**AML**	**AML-A**	**AML-B**	**AML-C***	**AML-D**
GCG	0.593	0.554	0.436[2]	0.581	0.417	0.639
AAG	0.357	0.251[1]	0.302	0.293	0.417	0.188[3]
ACG	0.028	0.080[4]	0.076[5]	0.063	0	0.106[6]
GAG	0.020	0.112[7]	0.186[8]	0.055	0.167	0.067[9]
AAA	0.002	0	0	0	0	0
GCA	0	0.003	0	0.008	0	0

[1] – **p=0.0018, OR=0.61, 95% CI (0.44-0.83) *vs.* C**

[2] – p=0.0094, OR=0.54, 95% CI (0.34-0.86) *vs.* C; p=0.0077, OR=0.45, 95% CI (0.25-0.81) *vs.* AML-D

[3] – **p=0.0011, OR=0.41, 95% CI (0.24-0.7) *vs.* C**

[4] – **p=0.001, OR=3.09, 95% CI (1.58-6.04) *vs.* C**

[5] – p=0.039, OR=2.82, 95% CI (1.05-7.56) *vs.* C

[6] – **p=0.0005, OR=4.32, 95% CI (1.9-9.81) *vs.* C**

[7] – **p<0.0001, OR=6.32, 95% CI (3.08-12.96) *vs.* C**

[8] – **p<0.0001, OR=11.28, 95% CI (4.87-26.13) *vs.* C**; **p=0.0048, OR=4.18, 95% CI (1.55-1.29) *vs.* AML-B;** p=0.022, OR=3.04, 95% CI (1.18-7.86) *vs.* AML-D

[9] – **p=0.0094 OR=3.71, 95% CI (1.38-10) *vs.* C**

* - AML-C group was not used for statistical analysis due to low number of patients in whom full genotypes were established (n=6)

Supplementary Table 2. Estimated frequency of reconstructed *FCN2* haplotypes (with EM maximum likelihood method) in patients and controls. C: controls; AML-A: patients who experienced infections with proven bacteremia and/or fungaemia; AML-B: patients who experienced infections with no bacteremia; AML-C: patients who experienced febrile neutropenia; AML-D: patients who experienced none of afore-mentioned complications within 4 weeks of hospital stay. Single nucleotide polymorphisms: -64 *A>C*, -4 *A>G*, +6359 *C>T* and +6424 *G>T* were used for reconstruction.

Haplotype	Group					
	**C**	**AML**	**AML-A**	**AML-B**	**AML-C***	**AML-D**
AACG	0.461	0.513	0.458	0.515	0.583	0.580[1]
AGTG	0.311	0.295	0.339	0.313	0	0.254
CACG	0.055	0.013	0.039	0	0	0
CACT	0.044	0.078	0.066	0.044	0.083	0.092
AACT	0.032	0.007	0	0.017	0.083	0
AATG	0.035	0.045	0.056	0.046	0	0.032
AATT	0.004	0	0	0	0	0
AGTT	0.030	0.003	0	0	0	0
AGCG	0.020	0.040	0.042	0.038	0	0.042
CGTG	0.005	0	0	0.009	0	0
CGCT	<0.001	0.005	0	0.017	0	0
CATT	0.003	0	0	0	0.083	0
CGTT	0	0	0	0	0.167	0

[1] – p=0.0291, OR=1.63, 95% CI (1.05-2.52) *vs.* C

* - AML-C group was not used for statistical analysis due to low number of patients in whom full genotypes were established (n=6)

Supplementary Table 3. Frequency of *MBL2* genotypes in patients (AML) and controls (C). Percentages are shown in parentheses

Genotype		Group	
		C (n=256)	AML (n=153)
A/A	***HYA/HYA***	36 (14.1)	15 (9.8)
	HYA/LYA	50 (19.5)	26 (17)
	HYA/LXA	34 (13.3)	28 (18.3)
	LYA/LYA	21 (8.2)	9 (5.9)
	LYA/LXA	24 (9.4)	14 (9.2)
	LXA/LXA	14 (5.5)	6 (3.9)
YA/O	***HYA/HYD***	5 (2)	3 (2)
	HYA/LYD	1 (0.4)	0
	HYA/LYB	29 (11.3)	8 (5.2)
	HYA/LYC	2 (0.8)	0
	LYA/HYD	3 (1.2)	3 (2)
	LYA/LYB	10 (3.9)	11 (7.2)
	LYA/LYC	1 (0.4)	1 (0.7)
	LYA/LYD	1 (0.4)	2 (1.3)
XA/O + O/O	***LXA/HYD***	6 (2.3)	6 (3.9)
	LXA/LYB	13 (5.1)	8 (5.2)
	LXA/LYC	0	3 (2)
	LXA/LYD	1 (0.4)	0
	LYB/HYD	0	3 (2)
	LYB/LYB	4 (1.6)	4 (2.6)
	LYB/LYC	1 (0.4)	2 (1.3)
	LYB/LYD	0	1 (0.7)

Supplementary Table 4. Frequency of *MBL2* haplotypes in patients and controls. C: controls; AML-A: patients who experienced infections with proven bacteremia and/or fungaemia; AML-B: patients who experienced infections with no bacteremia; AML-C: patients who experienced febrile neutropenia; AML-D: patients who experienced none of afore-mentioned complications within 4 weeks of hospital stay.

**Haplotype**	**Group**					
	**C**	**AML**	**AML-A**	**AML-B**	**AML-C***	**AML-D**
HYA	0.377	0.314	0.333	0.362	0.100	0.265[1]
LYA	0.256	0.248	0.256	0.241	0.400	0.235
LXA	0.207	0.234	0.269	0.190	0.200	0.255
HYD	0.027	0.050	0.013	0.043	0.100	0.059
LYD	0.006	0.010	0	0.017	0.100	0.010
LYB	0.119	0.135	0.128	0.129	0	0.167
LYC	0.008	0.020	0	0.017	0.100	0.010

[1] – p=0.032, OR=0.6, 95% CI (0.37-0.96) *vs.* C

* - AML-C group was not used for statistical analysis due to low number of patients in whom full genotypes were established (n=6)

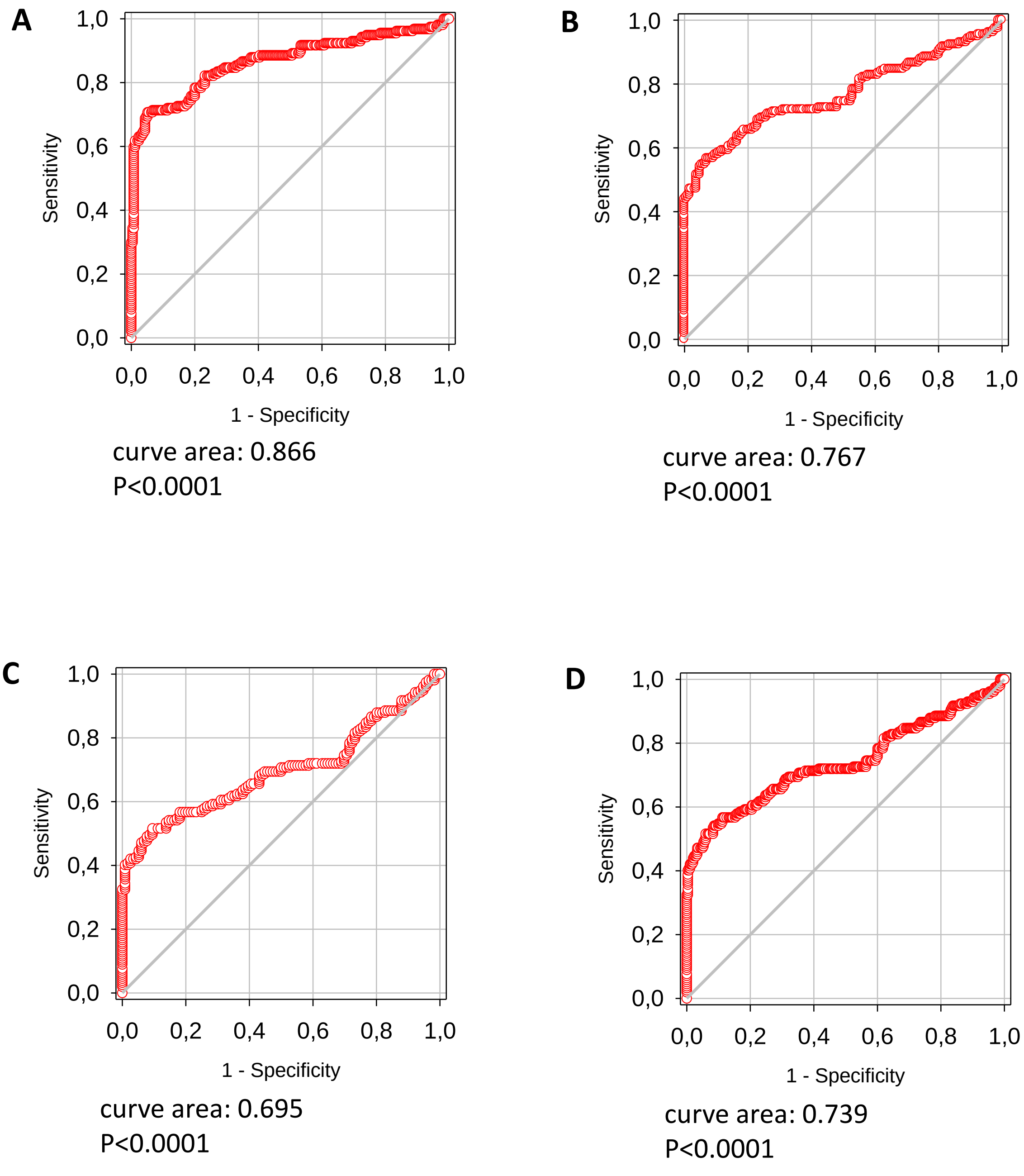

Supplementary Figure 1. Differentiation (ROC analysis) between patients suffering from acute myeloid leukaemia and healthy controls (A), multiple myeloma (B), lymphoma (C) and multiple myeloma plus lymphoma patients (D), using determination of ficolin-1 serum concentration.

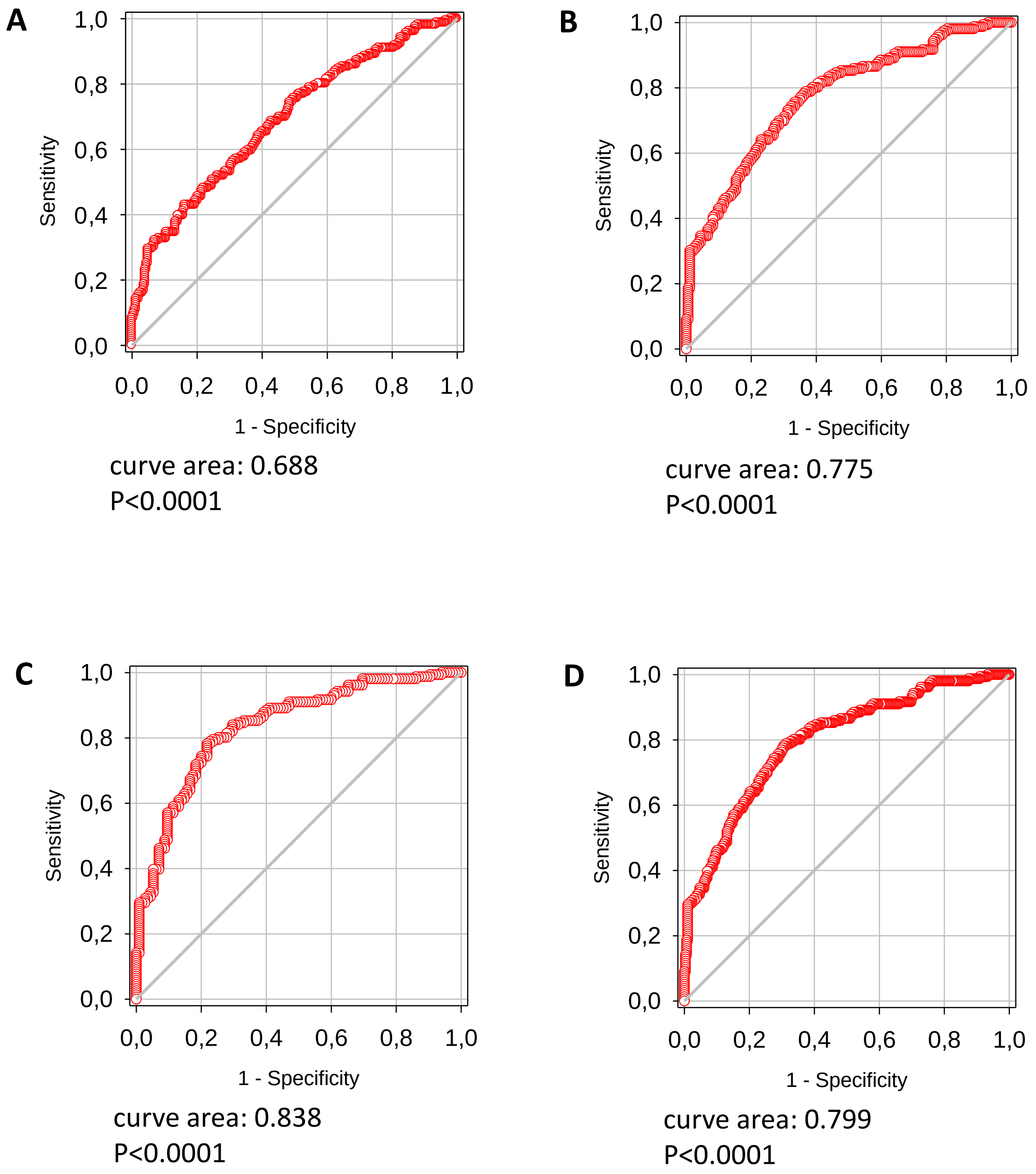

Supplementary Figure 2. Differentiation (ROC analysis) between patients suffering from acute myeloid leukaemia and healthy controls (A), multiple myeloma (B), lymphoma (C) and multiple myeloma plus lymphoma patients (D), using determination of ficolin-2 serum concentration.

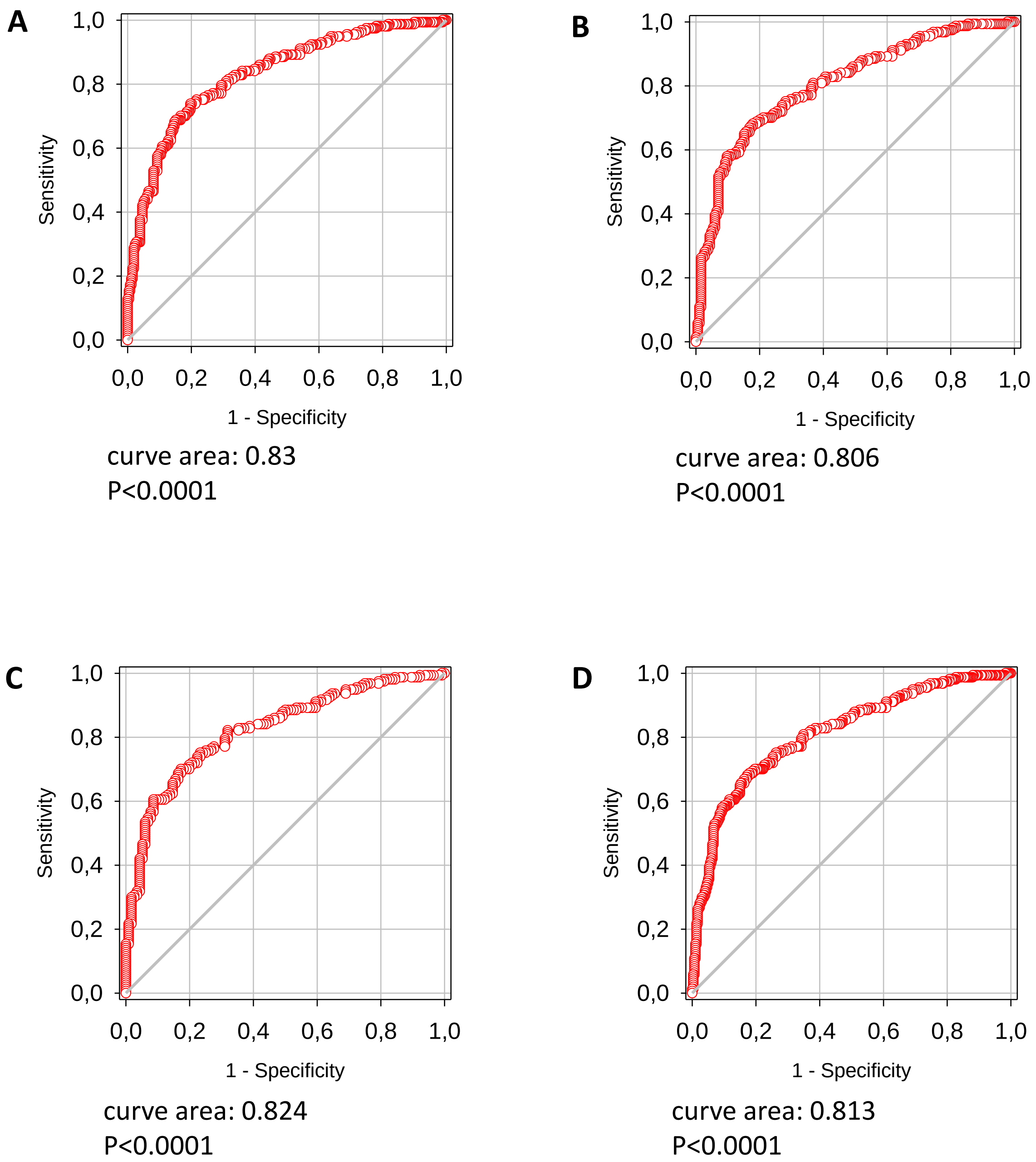

Supplementary Figure 3. Differentiation (ROC analysis) between patients suffering from acute myeloid leukaemia and healthy controls (A), multiple myeloma (B), lymphoma (C) and multiple myeloma plus lymphoma patients (D), using determination of ficolin-3 serum concentration.

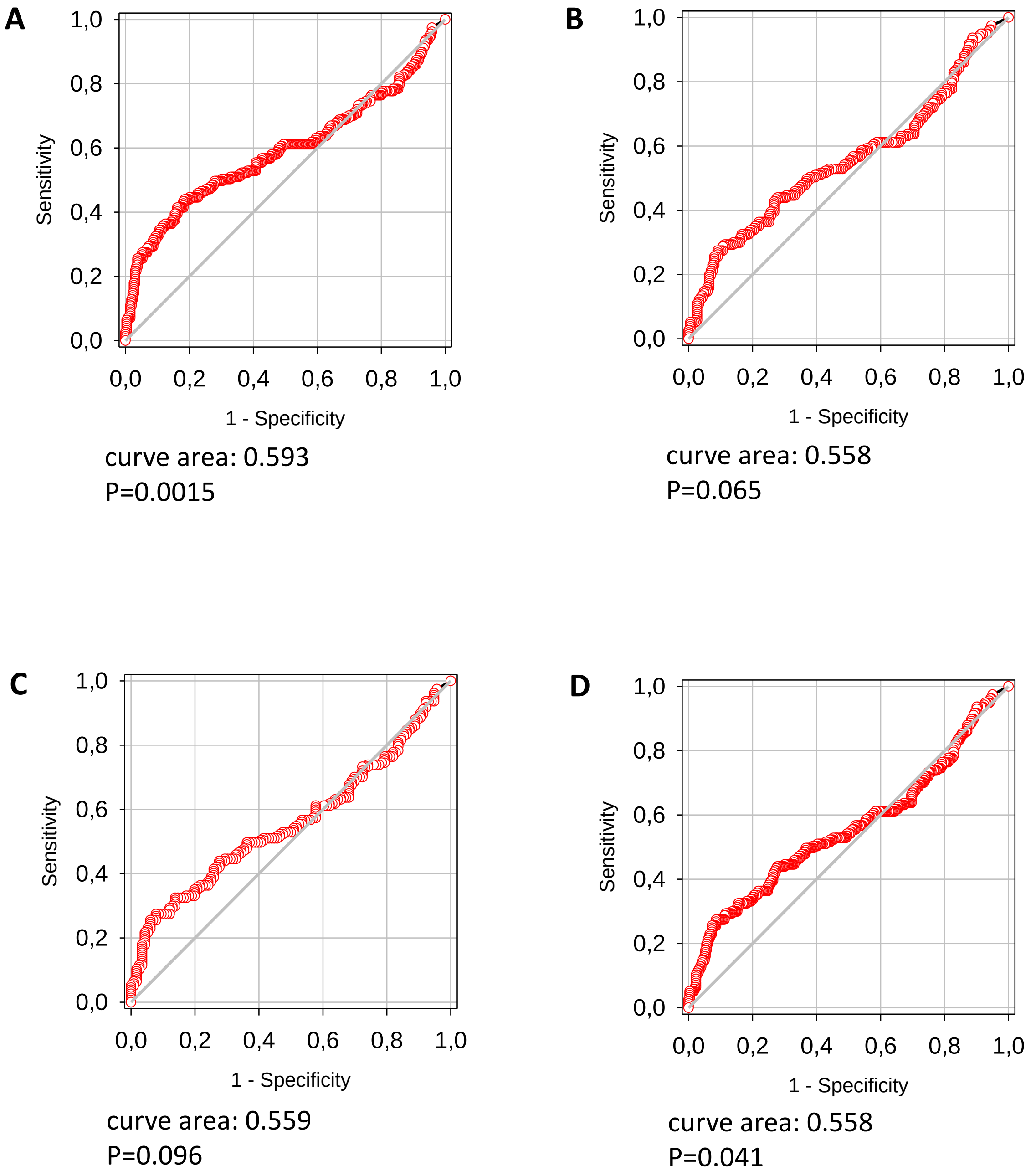

Supplementary Figure 4. Differentiation (ROC analysis) between patients suffering from acute myeloid leukaemia and healthy controls (A), multiple myeloma (B), lymphoma (C) and multiple myeloma plus lymphoma patients (D), using determination of mannose-binding lectin serum concentration.
